# Removal of Nitrate in Simulated Water at Low Temperature by a Novel Psychrotrophic and Aerobic Bacterium,* Pseudomonas taiwanensis* Strain J

**DOI:** 10.1155/2018/4984087

**Published:** 2018-03-28

**Authors:** Tengxia He, Qing Ye, Quan Sun, Xi Cai, Jiupai Ni, Zhenlun Li, Deti Xie

**Affiliations:** Chongqing Key Laboratory of Soil Multiscale Interfacial Process, Southwest University, Chongqing 400716, China

## Abstract

Low temperatures and high pH generally inhibit the biodenitrification. Thus, it is important to explore the psychrotrophic and alkali-resisting microorganism for degradation of nitrogen. This research was mainly focused on the identification of a psychrotrophic strain and preliminary explored its denitrification characteristics. The new strain J was isolated using the bromothymol blue solid medium and identified as* Pseudomonas taiwanensis* on the basis of morphology and phospholipid fatty acid as well as 16S rRNA gene sequence analyses, which is further testified to work efficiently for removing nitrate from wastewater at low temperature circumstances. This is the first report that* Pseudomonas taiwanensis* possessed excellent tolerance to low temperature, with 15°C as its optimum and 5°C as viable. The* Pseudomonas taiwanensis* showed unusual ability of aerobic denitrification with the nitrate removal efficiencies of 100% at 15°C and 51.61% at 5°C. Single factor experiments showed that the optimal conditions for denitrification were glucose as carbon source, 15°C, shaking speed 150 r/min, C/N 15, pH ≥ 7, and incubation quantity 2.0 × 10^6^ CFU/mL. The nitrate and total nitrogen removal efficiencies were up to 100% and 93.79% at 15°C when glucose is served as carbon source. These results suggested that strain J had aerobic denitrification ability, as well as the notable ability to tolerate the low temperature and high pH.

## 1. Introduction

Nitrate, due to its high water-soluble characteristic, is possibly the major nitrogen contaminant in the water [1, 2]. High concentration nitrate could contribute to water eutrophication [3] and even impose a serious threat to human health, such as malformation, carcinoma, and mutation when the nitrate transformed into nitrosamines [4]. The World Health Organization (WHO) has recommended that the nitrate concentration in drinking water should be lower than 10 mg/L [5] and the same value was also proposed by China [6]. Unfortunately, the nitrate concentration was higher than 10 mg/L in numerous aquifers and even exceeded 30 mg/L in some groundwater in China [7–9]. The nitrate remediation, thereby, is a big challenge and has become a matter of great concern in the recent years.

Previous researches have shown that the most commonly methods to remove nitrate from wastewater included biological denitrification with microorganism and physicochemical reduction using ion exchange, electrodialysis, reverse osmosis, zero-valent iron, and zero-valent magnesium [3, 10]. Several reports demonstrated that bioremediation has much higher efficiency, has lower cost, and is easier to implement compared with physicochemical methods for nitrate removal from wastewater [11, 12]. Likewise, numerous researchers discovered that the biological denitrification is the most promising approach because the bacteria could reduce the nitrate to the harmless nitrogen gas [13, 14]. Therefore, there are a host of papers dedicated to isolation and identification of nitrate reduction bacteria, such as* Stenotrophomonas* sp. ZZ15,* Oceanimonas* sp. YC13 [15],* Bacillus *sp. YX-6 [16],* Psychrobacter* sp. S1-1 [17],* Zoogloea *sp. N299 [18], and* Alcaligenes* sp. TB [19], while the reported denitrifying bacteria including these mentioned above are almost mesophilic bacteria and the optimum denitrification temperatures are between 20 and 35°C. However, these mesophilic denitrifying bacteria might face great challenges in winter months because the low temperatures generally drastically inhibit their denitrification ability, cell growth, and proliferation especially when the temperature was at 10°C or less [20, 21]. Thus, it is important to explore the bacteria which could effectively remove nitrate nitrogen at low temperatures.

Additionally, nitrate biodegradation may generate OH^−^ which could inhibit the denitrification process and enzyme activity [22]. Zhang et al. [23] discovered that neither cell density increase nor nitrate reduction was found if the pH is greater than 8.5; Li [22] reported that the nitrate reduction was completely inhibited when the pH reached about 9.5. Therefore, the optimal pH of most new isolated aerobic denitrifiers ranged from 6.5 to 7.0, such as* Ochrobactrum *sp. (6.5–7.0) [24],* Alcaligenes* sp. S84S3 (7.0) [25],* Psychrobacter* sp. (7.0) [17], and* Pseudomonas mendocina* 3–7 (7.0) [26]. It may be difficult for these microorganisms to meet the requirements of alkaline sewage treatment.

In this study, a novel bacterial strain for candidate of aerobic denitrifier, capable of aerobic denitrification with nitrate, was identified as* Pseudomonas taiwanensis*, named J. To the best of our knowledge, few nitrate denitrification studies have been performed to focus on the species of* Pseudomonas taiwanensis*. In this research, the impact resistance of strain J to low temperature, extreme alkalinity, and high C/N ratio were investigated using nitrate as sole nitrogen source. The experimental results showed that the strain J possessed excellent tolerance to low temperatures, with 15°C as its optimum and 5°C as viable. Furthermore, it has been found that the nitrogen removal efficiency did not decrease obviously with temperature between 15°C and 40°C. Significantly, high C/N ratio and strong alkalinity were not the limiting factors for cell density increase and nitrate denitrification performance. Accordingly, the strain J could be used to treat the high C/N ratio wastewater and the alkaline wastewater in four seasons.

## 2. Materials and Methods

### 2.1. Bacterium and Media

The psychrotrophic and aerobic denitrifying bacterium* Pseudomonas taiwanensis *strain J was stocked in 30% glycerol solution at −20°C.

We followed the methods of He and Li [27]. The bromothymol blue (BTB) solid medium per liter (pH = 7.2) comprised NaNO_3_ 1 g, KH_2_PO_4_ 1 g, FeCl_2_-6H_2_O 0.5 g, MgSO_4_-7H_2_O 1 g, CaCl_2_-7H_2_O 0.2 g, sodium succinate 8.5 g, BTB reagent 1 mL [1.5% in ethanol], and agar 20 g [28]. The modified denitrification medium (MDM) per liter comprised the following (ultrapure water, per liter): NaNO_3_ 0.31 g, CH_3_COONa 2.56 g, KH_2_PO_4_ 1.5 g, Na_2_HPO_4_ 0.42 g, MgSO_4_-7H_2_O 1 g, Fe SO_4_-7H_2_O 0.05 g, and pH 7.2 ± 0.1 [16]. LB medium (pH 7.0; per liter) included tryptone 10.0 g, yeast extract 5.0 g, and NaCl 10 g. For preparation of LB solid plates, 2% (w/v) agar powder was added.

### 2.2. Identification of the Psychrotrophic and Aerobic Denitrifying Bacterium Strain J

Colony morphologies of strain J were monitored on the BTB medium plates after incubating at 15°C for 3 d. Cell morphologies of strain J were observed under the HITACHI S-3000N scanning electron microscope and atomic force microscopy. Phospholipid fatty acids (PLFAs) were extracted by using about 40 mg pure culture of strain J after incubating 48 h at 15°C. Each type of PLFAs was analyzed by Agilent 6850.

The nearly full-length of 16S rRNA gene sequence was amplified using DNA as template which was extracted by genomic DNA purification kit (Thermo scientific). The universal primers 27F (5′-AGAGTTTGATCCTGGCTCAG-3′) and 1492R (5′-GGTTACCTTGTTACGACTT-3′) were used for polymerase chain reaction (PCR) amplification. The PCR amplification was conducted in the 50 mL volume containing 2 *μ*L DNA, 2 *μ*L primer, 25 *μ*L 2x Taq PCR Master Mix, and 19 *μ*L sterile water. The PCR conditions were denaturation for 5 min at 94°C, 30 cycles of 1 min at 94°C, 30 s at 55.5°C, and 1 min at 72°C and extension for 10 min at 72°C. The 1.5 kb product was separated on a 1.5% agarose gel and purified by BioSpin gel extraction kit (BioFlux). The purified product was cloned into pMD®20-T vector (Takara) and then sequenced by Invitrogen company. Then the 16S rRNA sequence of strain J was submitted to NCBI for accession number. Sequence alignment and multiple alignment were performed using NCBI Search Tool program (BLAST: https://blast.ncbi.nlm.nih.gov/Blast.cgi?PROGRAM=blastn&PAGE_TYPE=BlastSearch&LINK_LOC=blasthome) and CLUSTAL W. And the phylogenetic tree was constructed using MEGA 6.0 software by neighbor-joining distance method and bootstrap analyses of 1000 replicates.

### 2.3. Effects of Culturing Conditions on the Denitrification Ability of Strain J

Effects of six cultivation conditions including temperature (5°C, 10°C, 15°C, 20°C, 25°C, 30°C, 35°C, and 40°C), shaking speed (0 r/min, 50 r/min, 100 r/min, 150 r/min, and 200 r/min), pH (6.5, 7.0, 8.0, 9.0, and 10.0), inoculation quantity (0.5 × 10^8^ CFU, 1.0 × 10^8^ CFU, 1.5 × 10^8^ CFU, 2.0 × 10^8^ CFU, and 2.5 × 10^8^ CFU within 100 mL DM), and carbon source (sodium citrate, sodium succinate, sodium acetate, sucrose, and glucose) on denitrification performance of strain J were determined by the single factor tests. The amount of carbon was changed to adjust the C/N ratio to 0, 5, 10, 15, 20, and 25 by fixing the amount of NaNO_3_ as the nitrogen source. 1.0 × 10^8^ CFU precultured bacterial suspension was inoculated into 250 mL sterilized conical flask which contained 100 mL DM broth medium for testing, except for inoculation quantity which will be specified later. After incubating for 48 hours, nitrate and total nitrogen were then determined for these samples, in order to analyze the influences of the various factors indicated above.

The nitrate and total nitrogen removal efficiencies were calculated by the equation: Rv = (*T*_1_ − *T*_2_)/*T*_1_ × 100% to assess the denitrification ability of strain J. Note that Rv, *T*_1_, and *T*_2_ represent nitrate or total nitrogen removal efficiency, the initial concentration of nitrate or total nitrogen in MDM broth medium, and the final concentration of nitrate or total nitrogen in MDM broth medium after incubation for 48 hours, respectively. All experiments were conducted in triplicate.

### 2.4. Analytical Methods

The cell optical density (OD_600_) was monitored by measuring the absorbance at the wavelength of 600 nm using a spectrophotometer (DU800, BECKMAN COULTER, USA). Total nitrogen was calculated by the absorbance value at 220 nm subtracting the two times background absorbance value at 275 nm after alkaline potassium persulfate digestion. Nitrate was detected using the supernatant after samples centrifuged at 8000 rpm for 8 min. NO_3_^−^-N was calculated by the absorbance value at 220 nm subtracting the two times background absorbance value at 275 nm [29]. The value of pH was measured by pH-Meter (PHS-3D, Shanghai Precision and Scientific Instrument Corporation).

The final results were obtained by the average at least three independent experiments and were presented as means ± SD (standard deviation of means). All statistical analyses were carried out by one-way ANOVA with Tukey's HSD test (*P* < 0.05) using Excel and SPSS Statistics 22, and graphical works were carried out by Origin 8.6 software.

## 3. Results and Discussions

### 3.1. Identification of Strain J

**Figure 1:**
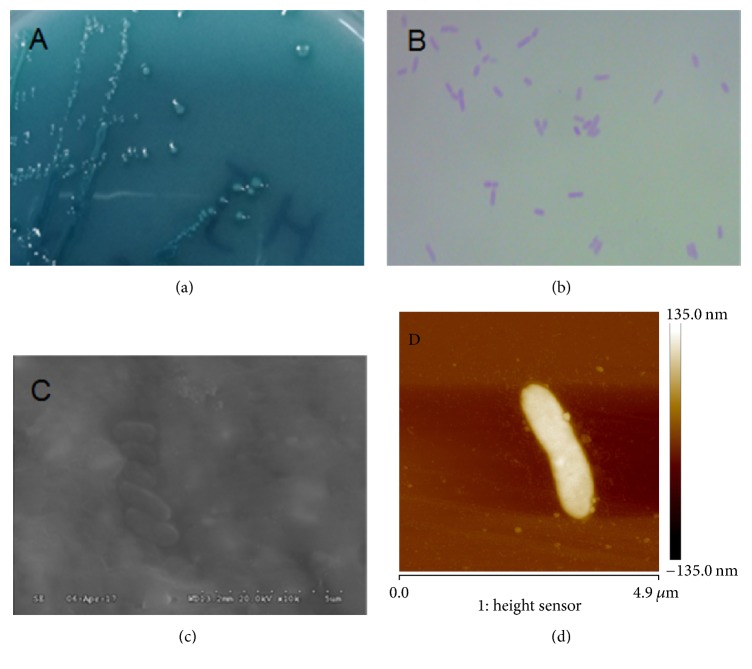
The morphologies of the strain J. Colony on BTB plates (a), unicell morphology of strain J (10 × 100) (b), cells under the scanning electron microscope (10,000x) (c), and atomic force microscopy (AFM) profile of strain J (d).

The colony morphologies of pure strain J are blue with a small white sport, convex, smooth with wet surfaces, regular edge, and opaque on BTB medium (Figure 1(a)). The strain J was gram-negative, rod-shaped, nonspore, and without flagellum (Figures 1(b), 1(c), and 1(d)).

Phospholipid fatty acids (PLFAs), as a key component of cell membrane, are an important indicator for identifying bacteria and fungi. The PLFAs is difference in different microbial groups but is relatively constant in the same microbial population, which have been proven to be relatively simple, fast, and inexpensive to be analyzed by gas chromatography [30, 31]. The PLFAs of strain J showed 0.627 similarity index with* Pseudomonas-syringae-syringae* in the version 6.0 of Sherlock® Microbial identification system. As depicted in Table 1, the value of 0.627 is greater than 0.5 which indicated that the strain J belongs to being a typical* Pseudomonas-syringae-syringae *according to the principle of PLFAs identification. However, the temperature, growth phase, carbon substrate, and so on maybe affect the PLFA composition. Therefore, we further identified the strain J by using 16S rRNA gene sequence which could enhance the credible of the identification result.

**Figure 2:**
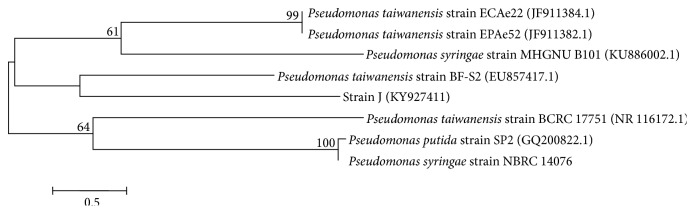
Phylogenetic tree of the strain J. Numbers in parentheses represent the sequences' in GenBank. The number at each branch point is percentage supported by bootstrap (1,000 resamplings). Bar: 0.1% sequence divergence.

The 16S rRNA gene sequence of strain J (1399 bp) was determined and exhibited 99% similarity with* Pseudomonas taiwanensis*. The phylogenetic tree was constructed by MEGA 6.0 software, which showed that the evolutionary divergence of strain J was also closely related to* Pseudomonas taiwanensis *instead of* Pseudomonas-syringae-syringae* (Figure 2). This difference identification results between PLFAs and 16S rRNA gene sequence might be that the version 6.0 of Sherlock Microbial identification system did not contain the species of* Pseudomonas taiwanensis*. Although the species of* Pseudomonas taiwanensis* has been reported by Volmer et al. [32] and Schmutzler et al. [33], whether the strain of* Pseudomonas taiwanensis* could conduct aerobic denitrification has not been researched. The accession number of strain J in GenBank nucleotide sequence databases is KY927411. Taking the physiological, phospholipid fatty acid and 16SrRNA gene sequence analyses into account, the strain J was identified as* Pseudomonas taiwanensis* and named J. Up to date, the species of* Pseudomonas taiwanensis* was hardly ever reported as the psychrotrophic aerobic denitrifier.

### 3.2. Effect of Temperature on Nitrogen Removal of the Strain J

**Figure 3:**
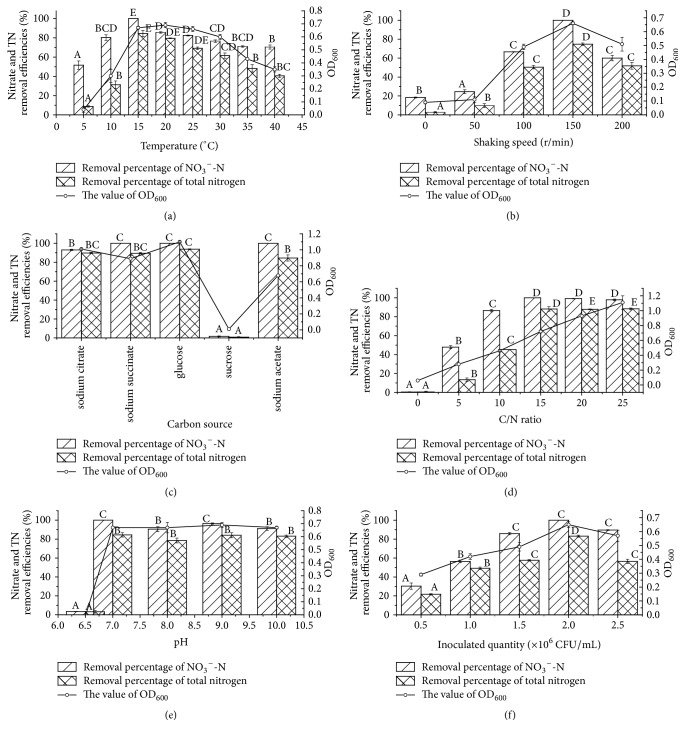
Growth of strain J and nitrate removal at different culturing conditions. Temperature (a), shaking speed (b), carbon source (c), C/N ratio (d), pH (e), and inoculated quantity (f). Values are means ± SD (errors bars) for three replicates.

Temperature is usually regarded as an environmental stress factor for the survival of bacteria because too high temperature could induce nucleic acid or protein denaturation, whereas too low temperature could result in inhibition of cell growth and proliferation, alter protein expression patterns, and weaken metabolic activity [34]. Generally, different bacteria have diverse temperatures for cell growth and functional activity. The effects of different temperatures on cell growth and nitrate removal of strain J in MDM medium were shown in Figure 3(a).* Pseudomonas taiwanensis *strain J was able to grow and conduct aerobic denitrification with nitrate as nitrogen source at a broad temperature range from 5 to 40°C, which may expand its application scope in different seasons. The strain J possessed tolerance to low temperature, with 15°C as its optimum and 5°C as viable, which suggested that strain J was a psychrotrophic aerobic bacterium. Data in Figure 3(a) showed that the nitrate nitrogen removal efficiency was enhanced with the increased temperature in a certain range. The nitrate and total nitrogen removal percentage increased from 51.61% and 8.76% at 5°C to 100% and 84.48% at 15°C under the condition of 150 r/min, with initial nitrate nitrogen concentration of about 50 mg/L after 48 h cultivation. These results demonstrated that higher temperature could promote the nitrate removal efficiency of strain J (*P* < 0.05). The nitrite nitrogen accumulation was distinctly observed at 5°C and 10°C with the concentrations of 15.0 and 20.12 mg/L, respectively (data were not shown in Figure 3). It was interesting that the nitrite-nitrogen was not detected when the temperature is higher than 15°C, or even below the nitrite detection limit when nitrate was used as sole nitrogen source. These results indicated that too low temperature might result in high nitrite accumulation. The similar results were reported that the nitrite could be accumulated as the temperature below 15°C [8, 35]. By contrast, the nitrate and total nitrogen removal efficiencies decreased continuously when the temperature further increased from 15°C to 40°C, and the nitrate and total nitrogen removal efficiencies decreased to 70% and 40.61% at 40°C, which demonstrated that the denitrification ability of strain J could be slightly inhibited by high temperatures. Accompanied with the reduction of nitrate and total nitrogen, the OD_600_ value of strain J increased firstly from 0.04 to 0.69, corresponding to an average growth rate of 0.345 d^−1^, and then decreased to 0.35 at 40°C. Above all results suggested that the temperature had a pronounced effect on the nitrogen removal efficiencies and cell growth of strain J (*P* < 0.05).

It is notable that nearly all the reported aerobic denitrifying bacteria were mesophiles and the optimum temperatures were ranged from 20 to 37°C, in which the cell growth and nitrogen denitrification were severely or totally inhibited at the temperature as low as 10°C [16, 36, 37]. As a result, the strain J could conduct aerobic denitrification efficiently at 5°C demonstrating that the strain J has much more tolerance to the cold condition, which may offer a new cold-adaptation aerobic denitrifier for nitrogen removal in winter.

### 3.3. Effect of Dissolved Oxygen on Nitrogen Removal of the Strain J

It has been widely accepted that the dissolved oxygen (DO) concentration in solution could be adjusted by the rotation speed of the shaker. The faster the shaking speed is, the higher the DO level can be obtained. In the aerobic denitrification process, both of the dissolved oxygen and nitrate nitrogen could act as electron accepters. A large amount of papers showed that a certain DO concentration was beneficial to bacterial growth and nitrogen removal [8, 38, 39]. The influence of different shaking speed on cell growth and nitrogen removal by strain J is shown in Figure 3(b). Significant differences were observed among shaking speed of 0–200 r/min (*P* < 0.05). Poor nitrogen removal efficiencies were obtained in static cultivation, with only 18.36% nitrate and 2.84% total nitrogen removal efficiencies after 48 h incubation. The higher rotation speed significantly promoted the cell reproduction and nitrogen removal efficiencies. The optimal shaking speed for strain J growth and denitrification was 150 r/min, which is equivalent to 6.25 mg/L DO concentration [37]. The peak of nitrate and total nitrogen removal efficiencies were 100% and 84.75% under the conditions of 150 r/min and 15°C. Subsequently, the cell growth and nitrogen removal ability were inhibited at the shaking speed of 200 r/min, which may be that the nitrate reductase is sensitive to high concentration of dissolve oxygen. The value of OD_600_ showed similar change with the nitrogen removal efficiencies and no obvious nitrite accumulation in the experiment. Apparently, the excessive low or high concentration of DO was unfavourable for bacterial growth and denitrification, which indicated that proper aeration could improve the nitrogen removal efficiencies of strain J in the practice application.

### 3.4. Effect of Carbon Source on Nitrogen Removal of the Strain J

Carbon sources, with different chemical structures and molecular weights, usually served as the electron donor and energy for heterotrophic aerobic denitrification process. Generally, carbon sources with simpler structures and low molecular weight were more beneficial for aerobic bacterial denitrification [19].  Figure 3(c) showed that the different carbon sources had markedly affected nitrogen reduction efficiencies (*P* < 0.05) at 15°C and 150 r/min. The experimental results showed that sodium citrate, sodium succinate, glucose, and sodium acetate could well support the growth of the strain J and promote nitrate reduction. Strain J exhibited the highest nitrogen removal ability when the glucose was used as the sole nitrogen source, with the nitrate and total nitrogen removal percentage of 100% and 93.79%, respectively. Glucose, due to its simple and small molecular structure, being the best carbon source for aerobic denitrification, was also discovered in the previous paper, such as the strain of* Anoxybacillus contaminans *HA [34]. Nevertheless, the sucrose was not good for both cell growth and nitrogen removal ability of strain J. The accumulation of nitrite nitrogen was not observed in this carbon source experiments. Therefore, it could be concluded here that a variety of carbon sources were beneficial for strain J conducting aerobic denitrification, implying that the carbon sources were not the limiting factors for strain J in the nitrate reduction process at low temperature.

### 3.5. Effect of C/N Ratio on Nitrogen Removal of the Strain J

Previous reports indicated that the high C/N ratio could promote the nitrogen reduction [40, 41]. Sodium succinate, located in central position of tricarboxylic acid cycle (TCA), was used as carbon source by fixing the nitrogen of NaNO_3_, which may provide electron donor and energy rapidly for cell multiplication and aerobic denitrification. The effects of different C/N ratio on cell growth and nitrogen removal by strain J were shown in Figure 3(d). The significant differences in nitrogen removal percentage and cell growth were obtained among C/N ratio of 0–15 (*P* < 0.05). Little nitrogen reduction and bacterial reproduction were observed when the C/N ratio was 0, suggesting that strain J was not an autotrophic denitrifier. With the C/N ratio increasing from 0 to 15, the nitrate and total nitrogen removal percentage reached the peak value of 100% and 88.24%. However, although C/N ratio further increased from 15 to 25, the removal efficiencies of nitrate and total nitrogen were comparatively constant, which manifested that although high C/N ratio could not accelerate nitrogen reduction, it also could not inhibit the nitrogen removal ability of strain J. The results dramatically differ from the previous studies that much higher C/N could lower the nitrogen removal efficiency. For instance, Zhang et al. [8] reported that the ammonium removal rate decreased at the C/N of 20 by* Microbacterium* sp. SFA13, and Huang et al. [18]. found that the nitrogen removal efficiency yielded a slight decrease as the C/N ratio higher than 2 with the strain of* Zoogloea* sp. N299. It might be concluded that the strain J could tolerate a much higher concentration of carbon source and the optimal C/N ratio was 15, which was consistent with the strain of* Marinobacter* sp. possessing the optimal C/N ratio of 15 [42]. Moreover, the bacterial proliferation increased continuously with increasing of C/N ratio, corresponding to a peak OD_600_ value of 1.11 at C/N ratio of 25, and no nitrite was detected in all C/N ratio solutions.

### 3.6. Effect of pH on Nitrogen Removal of the Strain J

The nitrate and total nitrogen removal using the strain J at different pH in the MDM was investigated with 150 r/min and 15°C, as shown in Figure 3(e). This figure depicted that the different pH also had a great effect on nitrogen reduction and bacterial growth (*P* < 0.05). The growth and denitrification activities of the strain J were almost unchanged at the initial pH value of 6.5, implying that slightly acidic was harmful to the cell reproduction and metabolic activities. But above pH 7, the nitrogen removal efficiencies and the value of OD_600_ were increased obviously. The maximum nitrate removal efficiency of 100% was obtained at pH 7.0. When the pH increased from 7.0 to 10.0, the nitrate removal percentage was lower than 100%, but higher than 90.51%. This result was consistence with the strain of* Acinetobacter* sp. CN86, in which the nitrate removal rate was lower slightly when the pH increased from 7.0 to 9.0 [43], but conflicted with another papers which reported that the nitrate reduction occurred under circumneutral pH or weak acidic conditions [44, 45]. Compared with the degradation of nitrate, the value of pH arranging from 7.0 to 10.0 had no distinctively effect on total nitrogen removal ability of strain J with the removal percentage about 84.0%. Meanwhile, all values of OD_600_ were almost equivalent to 0.67 except that the value of pH was 6.5. Apparently, the neutral and alkaline environments were conducive to heterotrophic aerobic denitrification, and the strong alkaline was not the limiting factor for aerobic denitrification.

### 3.7. Effect of Inoculation Quantity on Nitrogen Removal of the Strain J

The nitrate and total nitrogen removal efficiencies were directly affected by the amount of strain J (*P* < 0.05), as described in Figure 3(f). The aerobic denitrification percentage increased with the increase of the inoculation amount from 0.5 × 10^6^ CFU/mL to 2.0 × 10^6^ CFU/mL at 15°C. But further increase of incubation quantity to 2.5 × 10^6^ CFU/mL could result in a distinct decrease of the nitrogen removal efficiencies. The maximum nitrate and total nitrogen removal efficiencies were 100% and 83.15% and the maximum value of OD_600_ was 0.65 with inoculation amount of 2.0 × 10^6^ CFU/mL. It thus could be suggested that the cell growth space, oxygen supply, and nitrogen reduction might be influenced when inoculation quantity was higher than 2.0 × 10^6^ CFU/mL. Furthermore, there was no nitrite nitrogen accumulation in this inoculation quantity experiments. Thus, taking the nitrogen removal efficiencies and cell growth into consideration, the optimal inoculation quantity was 2.0 × 10^6^ CFU/mL. This optimized inoculation quantity of strain J could provide a reference for the actual wastewater treatment.

In this research, the nitrate nitrogen was used as the sole nitrogen source to preliminary explore whether strain J had a denitrification ability. The removal efficiencies of nitrate nitrogen and total nitrogen denoted the denitrification ability of the strain J, and the denitrification products (NO, N_2_O, and N_2_) should be further studied with nitrate as sole nitrogen source.

## 4. Conclusion

The novel psychrotrophic aerobic bacterium, named strain J, was identified as* Pseudomonas taiwanensis* based on morphology and phospholipid fatty acid as well as 16S rRNA gene sequence. Approximately 100.0% of nitrate nitrogen was removed at 15°C in different single factor experiment. The total nitrogen removal efficiency was higher than 83.15%. The strain J possessed excellent cold resistance and was able to remove nitrate at 5°C, with the removal percentage of 51.61%. The low temperature (below 15°C) was the only factor to result in nitrite accumulation. Most carbon sources could support aerobic denitrification except sucrose. Moreover, high C/N ratio and alkalinity condition were conducive to the cell density increase and nitrate denitrification.

## Figures and Tables

**Table 1:** The result of the specific PLFAs identification for strain J.

Library	Sim Index	Entry name
RTSBA66.21	0.627	*Pseudomonas-syringae-syringae*
	0.600	*Pseudomonas-fluorescens*-biotype B
	0.595	*Pseudomonas-fluorescens*-biotype A
	0.558	*Pseudomonas-putida*-biotype B/*vancouverensis*
	0.512	*Pseudomonas-syringae-glycinea*
	0.508	*Pseudomonas-putida*-biotype A
	0.485	*Pseudomonas-syringae-phaseolicola*
	0.480	*Pseudomonas-fluorescens*-biotype G/*taetrolens*
	0.452	*Herbaspirillum autotrophicum*
	0.425	*Pseudomonas-syringae-tabaci*
RCLIN66.20	0.581	*Chromobacterium-violaceum*
	0.489	*Pseudomonas-putida*-biotype A
MI7H103.80		(No match)

## References

[1] Guo L., Chen Q., Fang F. (2013). Application potential of a newly isolated indigenous aerobic denitrifier for nitrate and ammonium removal of eutrophic lake water. *Bioresource Technology*.

[2] Tang S., Yang Q., Shang H., Sun T. (2010). Removal of nitrate by autosulfurotrophic denitrifying bacteria: Optimization, kinetics and thermodynamics study. *Fresenius Environmental Bulletin*.

[3] Bhatnagar A., Sillanpää M. (2011). A review of emerging adsorbents for nitrate removal from water. *Chemical Engineering Journal*.

[4] Zhao Y., Feng C., Wang Q., Yang Y., Zhang Z., Sugiura N. (2011). Nitrate removal from groundwater by cooperating heterotrophic with autotrophic denitrification in a biofilm-electrode reactor. *Journal of Hazardous Materials*.

[5] Xing W., Li D., Li J., Hu Q., Deng S. (2016). Nitrate removal and microbial analysis by combined micro-electrolysis and autotrophic denitrification. *Bioresource Technology*.

[6] Su J. F., Ma M., Huang T. L. (2016a). Characteristics of autotrophic and heterotrophic denitrification by the strain pseudomonas sp. H117. *Geomicrobiology Journal*.

[7] Su J.-F., Shi J.-X., Huang T.-L., Ma F., Lu J.-S., Yang S.-F. (2016b). Effect of nitrate concentration, pH, and hydraulic retention time on autotrophic denitrification efficiency with Fe(II) and Mn(II) as electron donors. *Water Science and Technology A Journal of the International Association on Water Pollution Research*.

[8] Zhang D., Li W., Huang X., Wen Q., Miao L. (2013a). Removal of ammonium in surface water at low temperature by a newly isolated Microbacterium sp. strain SFA13. *Bioresource Technology*.

[9] Zhang X., Xu Z., Sun X., Dong W., Ballantine D. (2004). Nitrate in shallow groundwater in typical agricultural and forest ecosystems in China. *Journal of Environmental Sciences*.

[10] Sierra-Alvarez R., Beristain-Cardoso R., Salazar M., Gómez J., Razo-Flores E., Field J. A. (2007). Chemolithotrophic denitrification with elemental sulfur for groundwater treatment. *Water Research*.

[11] Leyva-Díaz J. C., González-Martínez A., González-López J., Muñío M. M., Poyatos J. M. (2015). Kinetic modeling and microbiological study of two-step nitrification in a membrane bioreactor and hybrid moving bed biofilm reactor-membrane bioreactor for wastewater treatment. *Chemical Engineering Journal*.

[12] Shrimali M., Singh K. P. (2001). New methods of nitrate removal from water. *Environmental Pollution*.

[13] Matějů V., Čižinská S., Krejčí J., Janoch T. (1992). Biological water denitrification-A review. *Enzyme and Microbial Technology*.

[14] Hauck R. D., Bouldin D. R. (1961). Distribution of isotopic nitrogen in nitrogen gas during denitrification. *Nature*.

[15] Yu L., Liu Y., Wang G. (2009). Identification of novel denitrifying bacteria Stenotrophomonas sp. ZZ15 and Oceanimonas sp. YC13 and application for removal of nitrate from industrial wastewater. *Biodegradation*.

[16] Song Z.-F., An J., Fu G.-H., Yang X.-L. (2011). Isolation and characterization of an aerobic denitrifying Bacillus sp. YX-6 from shrimp culture ponds. *Aquaculture*.

[17] Zheng H., Liu Y., Sun G., Gao X., Zhang Q., Liu Z. (2011). Denitrification characteristics of a marine origin psychrophilic aerobic denitrifying bacterium. *Journal of Environmental Sciences*.

[18] Huang T.-L., Zhou S.-L., Zhang H.-H., Bai S.-Y., He X.-X., Yang X. (2015). Nitrogen removal characteristics of a newly isolated indigenous aerobic denitrifier from oligotrophic drinking water reservoir, Zoogloea sp. N299. *International Journal of Molecular Sciences*.

[19] Chen J., Gu S., Hao H., Chen J. (2016). Characteristics and metabolic pathway of Alcaligenes sp. TB for simultaneous heterotrophic nitrification-aerobic denitrification. *Applied Microbiology and Biotechnology*.

[20] Rodriguez-Caballero A., Hallin S., Påhlson C., Odlare M., Dahlquist E. (2012). Ammonia oxidizing bacterial community composition and process performance in wastewater treatment plants under low temperature conditions. *Water Science and Technology*.

[21] Sundaresan N., Philip L. (2008). Performance evaluation of various aerobic biological systems for the treatment of domestic wastewater at low temperatures. *Water Science and Technology A Journal of the International Association on Water Pollution Research*.

[22] Li W. (2013). Study on characteristics in the removal process of ammonia nitrogen and nitrate nitrogen by an isolated heterotrophic nitrification-aerobic denitrification strain *Rhodococcus* sp. *Journal of Environmental Protection*.

[23] Zhang H., Wang H., Yang K., Sun Y., Tian J., Lv B. (2015). Nitrate removal by a novel autotrophic denitrifier (Microbacterium sp.) using Fe(II) as electron donor. *Annals of Microbiology*.

[24] Mahmood Q., Hu B., Cai J. (2009). Isolation of Ochrobactrum sp.QZ2 from sulfide and nitrite treatment system. *Journal of Hazardous Materials*.

[25] Angar Y., Kebbouche-Gana S., Djelali N.-E., Khemili-Talbi S. (2016). Novel approach for the ammonium removal by simultaneous heterotrophic nitrification and denitrification using a novel bacterial species co-culture. *World Journal of Microbiology and Biotechnology*.

[26] Zhu L., Ding W., Feng L.-J., Dai X., Xu X.-Y. (2012). Characteristics of an aerobic denitrifier that utilizes ammonium and nitrate simultaneously under the oligotrophic niche. *Environmental Science and Pollution Research*.

[27] He T.-X., Li Z.-L. (2015). Identification and denitrification characterization of a novel hypothermia and aerobic nitrite-denitrifying bacterium, Arthrobacter arilaitensis strain Y-10. *Desalination and Water Treatment*.

[28] Shao Q., Xiao-Bin Y.-U. (2008). Isolation and Characterization of A Strain Denitrobacteria. *Biotechnology*.

[29] APHA (2012). *Standard Methods for the Examination of Water and Wastewater*.

[30] Pratt B., Riesen R., Johnston C. G. (2012). PLFA Analyses of Microbial Communities Associated with PAH-Contaminated Riverbank Sediment. *Microbial Ecology*.

[31] Trögl J., Jirková I., Zemánková P. (2013). Estimation of the quantity of bacteria encapsulated in Lentikats Biocatalyst via phospholipid fatty acids content: A preliminary study. *Folia Microbiologica*.

[32] Volmer J., Neumann C., Bühler B., Schmid A. (2014). Engineering of Pseudomonas taiwanensis VLB120 for constitutive solvent tolerance and increased specific styrene epoxidation activity. *Applied and Environmental Microbiology*.

[33] Schmutzler K., Kracht O. N., Schmid A., Buehler K. (2016). Trophic regulation of autoaggregation in Pseudomonas taiwanensis VLB120. *Applied Microbiology and Biotechnology*.

[34] Chen J., Zheng J., Li Y., Hao H.-H., Chen J.-M. (2015). Characteristics of a novel thermophilic heterotrophic bacterium, Anoxybacillus contaminans HA, for nitrification–aerobic denitrification. *Applied Microbiology and Biotechnology*.

[35] Andersson A., Laurent P., Kihn A., Prévost M., Servais P. (2001). Impact of temperature on nitrification in biological activated carbon (BAC) filters used for drinking water treatment. *Water Research*.

[36] Zhang J., Wu P., Hao B., Yu Z. (2011). Heterotrophic nitrification and aerobic denitrification by the bacterium Pseudomonas stutzeri YZN-001. *Bioresource Technology*.

[37] Yao S., Ni J., Ma T., Li C. (2013). Heterotrophic nitrification and aerobic denitrification at low temperature by a newly isolated bacterium, Acinetobacter sp. HA2. *Bioresource Technology*.

[38] Ye J., Zhao B., An Q., Huang Y.-S. (2016). Nitrogen removal by Providencia rettgeri strain YL with heterotrophic nitrification and aerobic denitrification. *Environmental Technology (United Kingdom)*.

[39] Yu L., Wang Y., Liu H., Xi C., Song L. (2016). A novel heterotrophic nitrifying and aerobic denitrifying bacterium, Zobellella taiwanensis DN-7, can remove high-strength ammonium. *Applied Microbiology and Biotechnology*.

[40] Miqueleto A. P., Dolosic C. C., Pozzi E., Foresti E., Zaiat M. (2010). Influence of carbon sources and C/N ratio on EPS production in anaerobic sequencing batch biofilm reactors for wastewater treatment. *Bioresource Technology*.

[41] Kim M., Jeong S.-Y., Su J.-Y. (2008). Aerobic Denitrification of Pseudomonas putida AD-21 at Different C/N Ratios. *Journal of Bioscience and Bioengineering*.

[42] Zheng H.-Y., Liu Y., Gao X.-Y., Ai G.-M., Miao L.-L., Liu Z.-P. (2012). Characterization of a marine origin aerobic nitrifying-denitrifying bacterium. *Journal of Bioscience and Bioengineering*.

[43] Su J.-F., Shi J.-X., Huang T.-L., Ma F. (2016). Kinetic analysis of simultaneous denitrification and biomineralization of novel Acinetobacter sp. CN86. *Marine Pollution Bulletin*.

[44] Su J.-F., Zhang K., Huang T.-L., Wen G., Guo L., Yang S.-F. (2015). Heterotrophic nitrification and aerobic denitrification at low nutrient conditions by a newly isolated bacterium, Acinetobacter sp. SYF26. *Microbiology (United Kingdom)*.

[45] Wen Y., Wei C.-H. (2011). Heterotrophic nitrification and aerobic denitrification bacterium isolated from anaerobic/anoxic/oxic treatment system. *African Journal of Biotechnology*.

